# Evaluation of determining factors of early marriages and their impact on depressive symptoms: a study based on the Roma minority in Tirana, Albania

**DOI:** 10.1192/j.eurpsy.2025.1330

**Published:** 2025-08-26

**Authors:** E. Isuf

**Affiliations:** 1 Psychiatry, “Mother Teresa” UHC, Tirana, Albania

## Abstract

**Introduction:**

Early marriage is a widespread practice among the Roma population, an ethnic minority spread across Europe and other regions, including Albania. Despite efforts to address social disparities and promote human rights, early marriage persists and poses a significant challenge to the mental health and well-being of the individuals involved.

**Objectives:**

The study aims to evaluate the relationship between depressive symptoms and various determining factors that affect mental health within the Roma minority in Tirana as a result of early marriages. Specifically, it seeks to identify factors related to early marriage that impact individuals’ well-being, assess levels of depressive symptoms based on the total scores of the Beck Inventory, and examine depressive symptoms by gender. Additionally, the study will explore the relationship between socio-demographic data and depressive symptoms, as well as the connection between factors related to early marriage and depressive symptomatology, all measured through the total scores of the Beck Inventory.

**Methods:**

Our study is cross-sectional and examined 220 members of the Roma minority, residents of Tirana, both in urban and peripheral areas. This study explores the determining factors of mental health and their impact on depressive symptoms within the Roma community. The administered questionnaire consists of three parts, respectively covering the socio-demographic characteristics of the population, the Beck Inventory, and factors contributing to depressive symptoms.

**Results:**

The study included 220 Roma minority members, with 58.6% being female and 41.4% male. The average age of the subjects was 35.66 years, and 67.7% were married. Most participants lived in urban areas (65%), with the majority earning less than the minimum wage (40,000 ALL). Educationally, most had completed only primary education, and the average age at first marriage was 15.92 years, driven by love, tradition, and family pressure.

Depressive symptoms were reported by 60.5% of the subjects. Regression analysis identified statistically significant factors contributing to depressive symptoms, including age difference with the first partner, gender (with higher prevalence among females), education level, income level, and number of children. Other significant factors included satisfaction with education, loneliness, financial stress, and conflict frequency with partners.

**Conclusions:**

The analysis concluded statistically significant relationships (p≤0.05) between the sample and several determining factors in depressive symptoms.

This study can serve as a basis for more in-depth research aimed at developing more effective interventions and support strategies for this marginalized group.

**Disclosure of Interest:**

None Declared

